# Methodological and statistical concerns in MINERVA microbiome-disease knowledge graph

**DOI:** 10.1093/bib/bbaf673

**Published:** 2025-12-15

**Authors:** Salvatore Chirumbolo

**Affiliations:** Department of Engineering for Innovation Medicine, University of Verona, Strada Le Grazie 8, 37134 Verona, Italy

To the Editor,

Langarica et al. used a MINERVA AI and knowledge graphs to map microbe-disease links, enabling better research, analysis, and clinical decision-making [1].

MINERVA core functionality relies on constructing a knowledge graph (KG) *G* = (*V*, *E*), where nodes *V* represent microbes and diseases, and edges *E* ⊆ *V* × *R* × *V* represent directed relationships such as positive (promotive), negative (inhibitory), or unrelated associations. The edges are weighted according to a scoring function derived from multiple publications.

Actually, the paper emphasizes the platform's reliance on open-access data from PubMed and PubMed Central, which introduces a significant selection bias. By excluding paywalled articles, potentially high-quality research published in leading journals is systematically omitted. This leads to a skewed knowledge base that may overrepresent studies from certain regions, institutions, or fields where open-access publishing is more prevalent. Furthermore, the exclusion of grey literature and non-indexed datasets omits potentially valuable, albeit unconventional, sources of microbiome-disease insights. This selection process limits the diversity and scope of associations represented in the knowledge graph.

While this graph formalism is common in biomedical informatics, MINERVA methodology raises several issues in its instantiation of *V*, *E*, and the edge-weighting scheme. Confounding and bias in edge construction were observed.

Each edge *e_md_* = (*m*,*r*,*d*) connecting a microbe *m* ∈ *M* to a disease *d* ∈ *D* with relationship type *r* ∈{positive,negative,unrelated}, is derived from sentence-level relation extraction. This process is effectively a classification function:


(1) \begin{equation*} f:S\to R \end{equation*}

where *S* is a sentence containing both entities, and *R* is the relation label space. This is performed using a fine-tuned Large Language Model (LLM) with the following risk: the classification is made in isolation, ignoring document- or corpus-level evidence. The local context assumption:


(2) \begin{equation*} \forall s\in S:f(s)\approx \mathrm{True}\ \mathrm{Relation} \end{equation*}

is invalid in biomedical literature, where entities often require co-reference resolution and discourse-level interpretation to infer accurate relationships. For example, negations or hedging (e.g., “may”, “possibly”) alter semantic interpretation but are not explicitly modeled. Therefore, the assumption that each sentence-level prediction can be treated as an independent Bernoulli trial for relation classification is flawed:


(3) \begin{equation*} P\left(r|s\right)\ne P\left(r|\mathrm{document}\right)\ \mathrm{and}\kern0.5em \mathrm{Var}\left(f(s)\right)>\mathrm{acceptable}\ \mathrm{bounds} \end{equation*}

This inflates type I errors (false positives) in the graph.

Although the paper by Langarica et al. deserves attention [1], bias from data selection yet occurred.

Let *D*_Pub_ ⊂ *D*_True_ be the subset of all true microbiome-disease publications available in open-access repositories. If the true distribution of associations is *p*(*r,m, d*), MINERVA approximates this with $\hat{p}$ (*r, m, d*) using only *D*_Pub_. This introduces sampling bias:


(4) \begin{equation*} \hat{p\ }\left(r,m,d\right)=\frac{1}{\mid{D}_{\mathrm{Pub}}\mid}\sum_{i=1}^{\mid{D}_{\mathrm{Pub}}\mid }{I}_{r,m,d}^{(i)}\ \mathrm{with}\ {I}_{r,m,d}^{(i)}\in \left\{0,1\right\} \end{equation*}

But *D*_Pub_ is not i.i.d. sampled from *D*_True_, hence:


(5) \begin{equation*} KL\left(\hat{p\ }\left(r,m,d\right)|p\left(r,m,d\right)\right)\gg 0 \end{equation*}

indicating that MINERVA’s inferences are not representative of the true distribution of microbiome-disease relationships.

MINERVA employs a weighting scheme *w_md_* based on the journal impact factor *F_p_*, defined as:


(6) \begin{equation*} {w}_{md}=\sum_{k=1}^N{\operatorname{sign}}_{r_i}\cdotp{\log}_{10}\left({F}_{p_k}\right) \end{equation*}

This conflates publication prestige with experimental validity, violating the assumption that citation-level metadata correlates with relationship reliability:


(7) \begin{equation*} E\left[\mathrm{quality}|{F}_p\right]\approx \mathrm{high}\ \mathrm{variance} \end{equation*}

Furthermore, high-impact journals are subject to publication bias, often favoring novel or positive results, skewing *w_md_* toward overconfidence in certain associations and introducing systemic bias.

By investigating the relation prediction model evaluation metrics, it was noted that the link prediction model is a Graph Neural Network (GNN) with an *F*1 score of ~71%, implying that the model predictions have a non-negligible error margin. Let *y* ∈ {0,1} be the ground truth label and $\hat{y}$ the model prediction for an unobserved relation (*m, d*). Then:


(8) \begin{equation*} F1=\frac{2\cdotp \mathrm{precision}\cdotp \mathrm{recall}}{\mathrm{precision}+\mathrm{recall}}\approx 0.71 \end{equation*}

This corresponds to 29% misclassification, which, given the biological cost of false associations, renders the output suitable only for low-stakes exploratory analyses, not for clinical decision support. Moreover, the link prediction model uses Node2Vec embeddings, which rely on the graph’s structural proximity to infer similarity. However, in bioinformatics, structural similarity in a graph does not always imply functional or causal similarity. The embedding function *ϕ*: *V* → *R_d_* may preserve walk-based similarities but not biochemical or clinical relevance:


(9) \begin{equation*}
\left(\varphi \left({m}_1\right),\varphi \left({d}_1\right)\right)\approx \big(\mathrm{\varphi} \left({m}_2\right),\varphi \left({d}_2\right)\ne
\end{equation*}

shared biological mechanism

This undermines interpretability and scientific validity.

Moreover, there is confounding in risk score calculation.

The disease risk score is defined as:


(10) \begin{equation*} \mathrm{Risk}(d)=\sum_{i=1}^kI\left({m}_i\in \mathrm{deviant}\right)\cdotp \operatorname{sign}\left(r{m}_{d_i}\right) \end{equation*}

This simplistic summation assumes independence among microbial taxa, which violates ecological co-dependence. In microbial ecology, abundances are compositional (i.e. constrained by total sum):


(11) \begin{equation*} \sum_{i=1}^n{x}_i=1 \end{equation*}

Hence, increases in one taxon inherently imply decreases in others (compositional bias). Standard summative metrics on untransformed relative abundance data yield spurious associations, violating the principles of compositional data analysis (CoDA)**.** Proper risk scoring would require log-ratio transformations such as:


(12) \begin{equation*} {y}_i=\log \left(\frac{x_i}{g(x)}\right) \end{equation*}

where


$$ g(x)={\left(\prod_{j=1}^n{x}_i\right)}^{\frac{1}{n}} $$

without which, inferred disease risk profiles are statistically unsound.

Finally, there is no correction for multiple hypothesis testing.

The graph contains tens of thousands of edges, and multiple hypothesis tests are implicitly performed when evaluating microbe-disease associations [1]. However, the paper provides no mention of controlling for false discovery rate (FDR) using procedures like Benjamini-Hochberg. This omission leads to inflated type I errors:


(13) \begin{equation*} P\left(\mathrm{at}\ \mathrm{least}\ \mathrm{one}\ \mathrm{false}\ \mathrm{positive}\right)=1-{\left(1-\alpha \right)}^m\approx 1,\mathrm{when}\ m\gg 1 \end{equation*}

Without FDR correction, even high-confidence relations may be spurious in aggregate.

In summary, although MINERVA leverages advanced machine learning techniques and offers a user-friendly knowledge interface, its foundational assumptions, sentence-level independence, journal prestige as proxy for quality, absence of CoDA correction, and lack of multiple testing adjustment, are flawed from a bioinformatics and statistical perspective. To improve scientific validity, the platform must incorporate global textual context, compositional data transformations, rigorous FDR control, and probabilistic modeling of relation uncertainty.

**Key Points**

- MINERVA uses only open-access data, introducing strong selection bias.
- Sentence-level AI extraction ignores context, inflating false positives.
- Journal impact factor wrongly used as proxy for experimental validity.
- Risk score ignores compositional bias; lacks log-ratio data correction.
- No FDR control; model error undermines reliability.

## Data Availability

Any data to evaluate this Letter are available on request to the author.
